# A surgical approach to liposarcoma with retroperitoneal location: A case report and literature review

**DOI:** 10.1097/MD.0000000000042070

**Published:** 2025-04-25

**Authors:** Bowen Zou, Xuemei Wang, Jianchao Ma, Fagui Yue, Kaichuang Chen, Dongyang Luan, Xiaoliang Chen

**Affiliations:** a Urology Department, Jilin University Zhongri United Hospital, Changchun, China; b Pathology Department, Jilin University Zhongri United Hospital, Changchun, China; c Center for Reproductive Medicine and Center for Prenatal Diagnosis, First Hospital, Jilin University, Changchun, China.

**Keywords:** liposarcoma, retroperitoneal tumor, surgical resection

## Abstract

**Rationale:**

Liposarcoma with retroperitoneal location is a rare malignant tumor that poses significant diagnostic and therapeutic challenges due to its often asymptomatic nature in early stages and potential for substantial growth before detection.

**Patient concerns:**

We present the case of a 46-year-old female patient who experienced a 7-year history of intermittent left flank pain and was found to have a large, well-defined abdominal mass.

**Diagnosis:**

Urgent computed tomography scans revealed a retroperitoneal mass with characteristics suggestive of liposarcoma. Histopathological examination confirmed the diagnosis, showing features of both well-differentiated and dedifferentiated liposarcoma.

**Interventions:**

The patient underwent successful surgical resection of the mass, which measured 18 × 16 × 10 cm.

**Outcomes:**

The patient had an uneventful postoperative recovery and remained disease-free during the 6-month follow-up period, demonstrating the effectiveness of timely surgical intervention.

**Lessons:**

This case underscores the importance of accurate diagnosis and complete surgical resection in managing liposarcoma with retroperitoneal location. The high rate of local recurrence necessitates careful follow-up and consideration of adjuvant therapies. Further research is warranted to explore the optimal treatment strategies for recurrent cases.

## 1. Introduction

Liposarcoma with retroperitoneal location (RPLPS) is a malignant neoplasm that arises in the retroperitoneal space from mesenchymal tissue.^[1]^ It predominantly occurs in the retroperitoneum, particularly in perirenal adipose tissue, and often results in renal compression and displacement.^[2,3]^ RPLPS represents the most common soft tissue sarcoma found in this region, accounting for 0.07% to 0.2% of all tumors.^[1,3]^ Studies show that the incidence of liposarcoma increased by 19% from 2001 to 2016, with an annual growth rate of 1.43%. Among them, liposarcoma with retroperitoneal location showed a faster growth rate, with an annual growth rate of 1.94%, indicating that liposarcoma with retroperitoneal location is a high-risk subgroup.^[4]^ The disease primarily affects individuals aged 40 to 60 years, with a similar incidence in both sexes.^[5]^ The average age of liposarcoma patients is 61,^[6]^ while the median age of liposarcoma with retroperitoneal location patients in Asia is 57, which is younger than the 63 years seen in Western populations, suggesting that this disease may be more common in younger populations.^[7]^

In its early stages, liposarcoma with retroperitoneal location typically presents asymptomatically, as the expansive retroperitoneal space allows for undetected tumor growth.^[8]^ When symptoms do manifest, the average tumor size is approximately 15 cm,^[9]^ with half of the diagnosed liposarcomas exceeding 20 cm in diameter, posing significant therapeutic challenges.^[1,3]^ According to the World Health Organization’s classification of soft tissue and bone tumors, liposarcoma is categorized into well-differentiated and dedifferentiated subtypes.^[8]^ Recent genomic studies have further divided this disease into 4 distinct types: well-differentiated liposarcoma (WDL), dedifferentiated liposarcoma (DDL), myxoid liposarcoma (MLS), and pleomorphic liposarcoma (PLS).^[5,10]^ WDL is the most common subtype, typically characterized by indolent growth. However, it has a relatively high local recurrence rate and is often resistant to both radiotherapy and chemotherapy.^[11,12]^ The development of WDL is commonly associated with amplifications on chromosome 12, leading to the overexpression of disease-driving genes.^[11]^ DDL represents a more aggressive progression of WDL, characterized by increased invasiveness, a higher propensity for metastasis, and a poorer prognosis.^[11,12]^ Like WDL, DDL also involves amplifications on chromosome 12.^[11]^ MLS is marked by characteristic chromosomal translocations that result in the formation of oncogenic fusion proteins.^[11]^ PLS is the most aggressive subtype, with a complex karyotype and a high degree of genetic heterogeneity, contributing to its poor prognosis.^[13]^

Surgical resection remains the primary treatment modality for primary RPLPS and is considered potentially curative.^[1–3,5,8–10,14–19]^ However, due to the high rate of local recurrence, chemotherapy has emerged as the principal treatment for recurrent or advanced retroperitoneal sarcomas.^[8]^ For unresectable tumors, neoadjuvant therapies, including radiotherapy and chemotherapy, are considered feasible options.^[5]^

The primary objective of this study is to critically evaluate the clinical characteristics, diagnostic methodologies, surgical management, and postoperative outcomes of RPLPS, with a specific focus on addressing the unique challenges it presents in clinical practice. Given the rarity of RPLPS and its often asymptomatic nature in the early stages, the study seeks to underscore the pivotal role of early detection and timely surgical intervention in improving patient prognosis. Furthermore, the research aims to explore the high recurrence rates associated with this malignancy, offering insights into potential management strategies for recurrent cases, including the application of neoadjuvant therapies and the importance of achieving clear surgical margins. By contributing to the existing body of knowledge, this study aims to enhance clinical decision-making processes and inform the development of optimized treatment protocols. Ultimately, the findings of this study may facilitate improved patient outcomes and provide a valuable foundation for the evolution of treatment paradigms for liposarcoma with retroperitoneal location.

## 2. Case presentation

This study was approved by the Ethics Committee of the China-Japan Union Hospital of Jilin University and informed written consent was obtained from the patient for publication of this case report.

### 2.1. Clinical presentation

**Figure 1.**
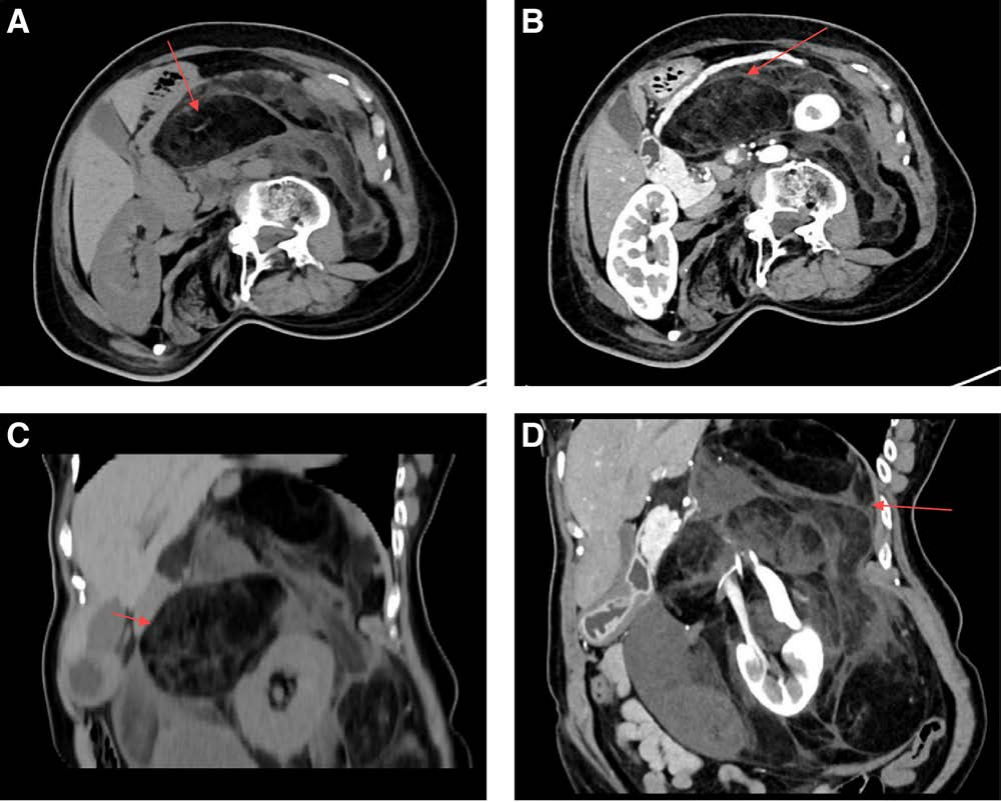
(A) Axial CT scan demonstrates a large retroperitoneal mass with heterogeneous density, causing displacement of adjacent structures. (B) Contrast-enhanced CT reveals the mass with mild delayed enhancement, further suggesting a malignant process. (C and D) The liposarcoma can be seen encircling the kidney on both non-contrast and contrast-enhanced CT scans. (D) heterogeneous enhancement of the liposarcoma tissue is observed.

A 46-year-old female patient presented on November 6, 2023, with a 7-year history of intermittent left flank pain. The patient reported episodes lasting from a few minutes to several tens of minutes, often relieved by oral analgesics, with no notable change in body weight. There was no family history of hereditary diseases. Physical examination revealed a large, painless, well-defined mass occupying the entire abdomen and associated spinal scoliosis. Laboratory tests showed no significant abnormalities. Computed tomography (CT) scans of the adrenal glands and kidneys, both plain and enhanced, revealed no enlarged lymph nodes in the retroperitoneum, but identified an irregularly shaped mixed-density mass with unclear borders, CT values ranging from -94 to 22 HU, and uneven mild delayed enhancement. The left kidney was enveloped by the lesion. The CT findings suggested a retroperitoneal mass with a high probability of liposarcoma (Fig. 1). The preoperative diagnosis was confirmed as a retroperitoneal tumor, and the patient subsequently underwent resection of the mass. The surgical procedure was uneventful, with an estimated blood loss of approximately 300 mL.

### 2.2. Postoperative specimen appearance

The postoperative specimen consisted of an irregularly shaped mass measuring 18 × 16 × 10 cm. The tumor exhibits an incomplete capsule with local infiltration. The cut surfaces exhibited a mix of grayish-white and pale yellow areas, with some regions showing a mucoid appearance and a fine, relatively smooth texture. These features, including the lack of a well-defined capsule and the presence of necrosis and cystic degeneration, are consistent with the typical characteristics of liposarcoma with retroperitoneal location.

### 2.3. Microscopic examination

#### 2.3.1. Histopathological description

The tumor is of lipogenic origin, composed of variably sized mature adipocytes, multivacuolated lipoblasts, and atypical bizarre stromal cells. The stromal cells exhibit enlarged, hyperchromatic nuclei with significant atypia. Focal myxoid changes and collagen deposition are observed in the stroma, accompanied by numerous small blood vessels and patchy inflammatory infiltrates. No definitive necrosis or mitotic figures are identified.

#### 2.3.2. Immunohistochemical results

MDM2 (+), CDK4 (+), P16 (+), S-100 (+), Ki67 (10% +), CD34 (+), H3K27Me3 (+), Rb (+),DDIT3 (‐), SMA (‐), STAT6 (‐) (Table 1).

**Table 1:** Immunohistochemistry results and antibody information.

Antibody	Result	Clone	Manufacturer	Country of origin
MDM2	+	OTI17B3	ZSJB-BIO	China
CDK4	+	EP180	ZSJB-BIO	China
P16	+	1C1	ZSJB-BIO	China
S-100	+	15E2E2	ZSJB-BIO	China
Ki67	10% +	UMAB107	ZSJB-BIO	China
CD34	+	EP88	ZSJB-BIO	China
SMA	–	UMAB237	ZSJB-BIO	China
STAT6	–	EP325	ZSJB-BIO	China
H3K27Me3	+	RM175	ZSJB-BIO	China
Rb	+	13A10	ZSJB-BIO	China
DDIT3	–	9C8	MXB	China

Antibodies were sourced from ZSJB-BIO (China) for MDM2 to Rb and MXB (China) for DDIT3, with staining results categorized as positive (staining present) or negative (no staining), and Ki67 showing 10% positivity; immunohistochemistry was performed on formalin-fixed, paraffin-embedded tissue using the EnVision system (DAB chromogen).

#### 2.3.3. Diagnostic conclusion

**Figure 2.**
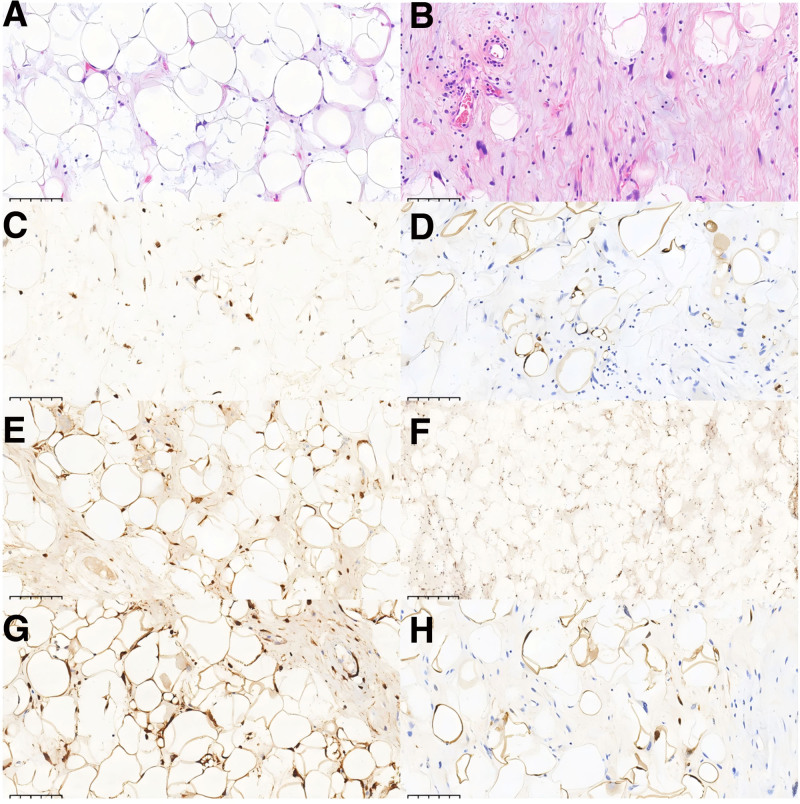
(A) Hematoxylin and eosin staining shows lipoblasts, original magnification 200×. (B) Hematoxylin and eosin staining reveals spindle mesenchymal cells with bizarre nuclei. Original magnification 200×. (C) Immunohistochemistry demonstrates strong positivity for MDM2, original magnification 200×. (D) Immunohistochemistry demonstrates positivity for CD34, original magnification 200×. (E) Immunohistochemistry demonstrates strong positivity for CDK4, original magnification 200×. (F) Immunohistochemistry demonstrates positivity for Rb, original magnification 100×. (G) Immunohistochemistry demonstrates positivity for P16, original magnification 200×. (H) Immunohistochemistry demonstrates positivity for S-100, original magnification 200×.

The histomorphological features and immunohistochemical profile support the diagnosis of liposarcoma, demonstrating characteristics of both well-differentiated and dedifferentiated components (Fig. 2). The Ki67 proliferation index is 10% in hotspot areas, while CD34 is negative in spindle cells. MDM2 and CDK4 show positive nuclear expression in lipoblasts. Based on the scoring system reported by Deacu M et al., the tumor scored 6 points.^[20]^ The patient did not undergo FISH testing post-surgery. The patient recovered uneventfully postoperatively.

### 2.4. Follow up

Following the surgery, the patient was closely monitored and underwent regular follow-up assessments. At both the 3-month and 6-month follow-ups, the patient was found to be in stable condition, with no significant abnormalities identified during clinical examinations or imaging studies. The patient reported no new symptoms and experienced no complications throughout the recovery process. We will continue to ensure the patient’s well-being through scheduled follow-up appointments.

## 3. Discussion

Liposarcoma with retroperitoneal location typically presents asymptomatically in its early stages, with most tumors discovered incidentally, often due to abdominal distension, indigestion, respiratory difficulties, or routine abdominal examinations.^[5]^ This case presented with a large, painless, well-defined abdominal mass and a history of nonspecific low back pain for 7 years, which aligns with typical manifestations of liposarcoma with retroperitoneal location.

CT scans are the diagnostic gold standard for evaluating liposarcoma with retroperitoneal location.^[1–3,5,8–10,14–19,21–24]^ They assist physicians in determining the tumor’s location, size, and its relationship to surrounding tissues and organs. Tumor characteristics are revealed through CT imaging; for instance, low-grade malignant tumors typically appear fat-like and radiolucent, while intermediate and high-grade tumors appear denser and more heterogeneous. CT imaging is crucial for surgical planning, allowing surgeons to ascertain the precise tumor location and key structures involved, thereby facilitating thorough surgical preparation. Additionally, CT scans are employed to monitor for tumor recurrence postoperatively, typically occurring within 6 months to 2 years after the procedure.^[3]^ Magnetic resource imaging (MRI) provides detailed visualization of tumor margins, helping surgeons to better understand the tumor’s relationship with adjacent organs, tissues, and vessels. Furthermore, MRI aids in surgical planning by allowing for precise three-dimensional reconstructions preoperatively, thereby enhancing the selection of optimal surgical approaches and resection scope.^[5]^

Preoperative fine needle aspiration cytology (FNAC) and biopsy play crucial roles in clarifying diagnosis and treatment planning.^[5]^ For patients with distant metastases or unresectable tumors, histological biopsy is recommended to guide subsequent therapeutic strategies. For large tumors that cannot be fully excised in one surgery, biopsy results are critical for determining further treatment directions. In resectable cases, biopsy can provide precise histological classifications, assisting surgeons in planning complete resections. Some patients may require neoadjuvant therapy to downstage tumors before surgery; here, biopsy information is essential for selecting neoadjuvant treatment regimens. For giant tumors, FNAC and biopsy can provide cytological characteristics, assisting physicians in more accurately diagnosing and managing treatment. Overall, preoperative FNAC and biopsy techniques serve as auxiliary and guiding tools in the diagnosis and treatment of liposarcoma with retroperitoneal location, helping clinicians better understand tumor characteristics and make informed treatment decisions.^[5]^

Surgical resection remains the mainstay treatment for RPLPS, particularly for locally recurrent tumors.^[1–3,8–10,14–19,24]^ The objective of surgery is to achieve negative microscopic margins.^[5]^ However, due to anatomical constraints, RPLPS may be asymptomatic in its early stages and is often detected only when the tumor has grown sufficiently large to compress adjacent organs. Tumors may develop large and deep, complicating resection. The size of tumors can complicate anatomical relationships, making it challenging to identify surgical margins and protect surrounding vessels and ureters. Surgeons may lack experience in handling such large tumors. Complete resection is crucial for treating this disease; otherwise, the risk of recurrence is significantly elevated. Achieving wide resection margins can be challenging, contributing to high local recurrence rates. In cases where the tumor invades adjacent organs, multi-organ resection may be necessary.^[19]^ Sosnowska-Sienkiewicz et al., have reported successful laparoscopic surgeries for dedifferentiated liposarcoma with osteosarcomatous differentiation, with follow-up CT showing no local or distant recurrence after 1.5 years.^[9]^ Research by Xiaoyi Wei and Antonino Agrusa demonstrated effective laparoscopic treatment of liposarcoma with retroperitoneal location, achieving complete tumor resection without recurrence during follow-up. This indicates that laparoscopic surgery can be effectively employed to treat liposarcoma with retroperitoneal location under appropriate clinical circumstances.^[17,21]^ Currently, there is no consensus regarding the surgical resection margins for RPLPS.^[2]^

For tumors that cannot be surgically removed, neoadjuvant treatments, including radiotherapy and chemotherapy, are viable options. Neoadjuvant therapy may reduce tumor size and stage, facilitating complete resection and increasing the likelihood of achieving negative margins.^[5]^ However, some studies suggest that preoperative radiotherapy does not prolong disease-free survival compared to surgery alone and may exacerbate adverse effects.^[17]^ Liposarcoma is relatively sensitive to radiotherapy, indicating it may help mitigate local recurrence risk, though there is insufficient evidence regarding its significant benefits. Chemotherapy or radiotherapy may be considered for unresectable or metastatic tumors, though these treatments generally exhibit limited efficacy.^[10]^

At present, targeted therapies for postoperative liposarcoma with retroperitoneal location show no significant advantages. Despite treatments based on common disrupted molecular pathways in RPLPS lacking sufficient therapeutic responses, ongoing research into the molecular mechanisms of this disease aims to identify novel therapeutic targets.^[22]^ In recent years, significant advances have been made in the genomic characterization of RPLPS. A genomic study has provided an overview of the genetic distinctions across different subtypes, with particular emphasis on the genomic heterogeneity of the disease. RPLPS is categorized into 4 main subtypes: WDL, DDL, MLS, and PLS. In WDL and DDL, beyond the well-known MDM2/CDK4 pathway, other potential therapeutic targets, such as FRS2, have been identified. For MLS, novel pathways, including YAP-1, have been proposed as potential targets for treatment.^[10]^

WDL usually consists of mature adipose tissue, with fibrous septa and variable nuclear atypia and enlargement.^[25]^ Microscopically, the tumor cells in WDL resemble normal adipocytes but exhibit fibrous septa and mild cellular atypia.^[26]^ DDL, on the other hand, is characterized by undifferentiated, pleomorphic, or spindle-shaped sarcoma cells, often adjacent to WDL or as a recurrence or de novo lesion.^[25,27]^ Microscopically, DDL shows a sudden transition from lipomatous areas (WDL) to non-lipomatous, high-grade regions containing significant mitotic activity.^[26]^ DDL can be differentiated from other undifferentiated sarcomas by detecting amplifications of the MDM2 and CDK4 genes, which serve as characteristic molecular markers.^[25,28]^ Immunohistochemical markers such as MDM2 and p16 can distinguish WDL from lipomas, with WDL showing positivity for these markers, while lipomas are typically negative.^[29]^ Additionally, angiomyolipomas can be confused with WDL/DDLS, but angiomyolipomas usually expresses HMB45 and Melan-A, which are not expressed in WDL/DDLS.^[29]^

In this case, the patient underwent successful resection of a retroperitoneal tumor, with an estimated intraoperative blood loss of 300 mL. Histopathological examination confirmed the diagnosis of liposarcoma, displaying both well-differentiated and dedifferentiated features. No adjuvant radiotherapy or chemotherapy was administered postoperatively, and the patient remained disease-free during the 6-month follow-up period.

Liposarcoma with retroperitoneal location typically recurs within 6 months to 2 years following initial surgical resection.^[5]^ The risk of recurrence is influenced by factors such as histologic subtype,^[8,15,18,24]^ surgical margin status, and the completeness of tumor resection.^[5]^ Local recurrence rates are notably higher in DDLPS and PLS.^[5]^ Achieving negative (R0) surgical margins is crucial for improving prognosis.^[19]^ Furthermore, PLS and myxoid pleomorphic liposarcoma (MPLPS) demonstrate a higher propensity for distant metastasis, which significantly impacts long-term outcomes.^[5]^ Lymph node involvement and distant metastasis are key prognostic factors.^[3]^ Current guidelines recommend follow-up every 3 to 6 months during the first 2 to 3 years postoperatively, transitioning to biannual follow-ups thereafter, with annual surveillance after 2 years.^[3]^

Given the known sensitivity of liposarcoma with retroperitoneal location to radiotherapy, future treatment approaches may focus on optimizing radiation therapy protocols, including exploring different regimens and dosages to improve therapeutic outcomes. While current evidence does not support the significant efficacy of chemotherapy for liposarcoma with retroperitoneal location, ongoing research may identify more effective chemotherapeutic agents or combinations. Furthermore, additional basic research is crucial to gaining a deeper understanding of the pathogenesis of liposarcoma with retroperitoneal location, which could lead to the development of novel therapeutic strategies. With the increasing use of laparoscopic surgery in managing retroperitoneal tumors, the potential for minimally invasive techniques is promising. For this patient, long-term follow-up is essential to monitor for recurrence or complications, enabling timely intervention if necessary. Concurrently, with the utilization of genetic testing technology and the progress in molecular biology, it is expected that upcoming research will result in the emergence of specific targeted therapies for liposarcoma with retroperitoneal location.

## 4. Conclusion

This case underscores the importance of early and accurate diagnosis of RPLPS, particularly given its often asymptomatic nature in the early stages. The key lessons learned from this case include the critical role of imaging, especially CT and MRI, in determining tumor location, size, and its relationship to surrounding structures, which are vital for preoperative planning and surgical approach. The absence of a well-defined capsule and the infiltrative nature of the tumor presented significant challenges during resection, emphasizing the necessity of achieving negative surgical margins to reduce the risk of local recurrence. Furthermore, this case highlights the value of histopathological and immunohistochemical analysis in confirming the diagnosis and differentiating RPLPS from other similar tumors, such as lipomas and angiomyolipomas. Lastly, the importance of careful follow-up is emphasized, as liposarcoma with retroperitoneal locations are prone to recurrence, requiring long-term monitoring for potential metastasis or local recurrence.

## Author contributions

**Investigation:** Xuemei Wang, Kaichuang Chen.

**Methodology:** Jianchao Ma, Dongyang Luan.

**Writing – review & editing:** Xiaoliang Chen, Fagui Yue.

**Writing – original draft:** Bowen Zou.
